# Effect of pomegranate peel powder extract (ethanolic) on performance, egg quality, thyroid hormones, antioxidant parameters, interferon γ, lipid and protein profiles of aged laying hens

**DOI:** 10.1007/s11250-025-04736-6

**Published:** 2025-12-10

**Authors:** Ramadan Allam Sayed AbdElrasoul, Samah Mohamed Youssef, Randa Ahmed Deif-Allah, Marwa Hosni Abd El-Maged

**Affiliations:** 1 Poultry Production Department, Faculty of Agriculture, Fayoum University, Fayoum, 63514 Egypt; 2 Horticulture Department, Faculty of Agriculture, Fayoum University, Fayoum, 63514 Egypt; 3 Poultry Nutrition Department, Animal Production Research Institute, Agricultural Research Center, Dokki, Giza Egypt; 4 Poultry Breeding Department, Animal Production Research Institute, Agricultural Research Center, Dokki, Giza Egypt

**Keywords:** Aged laying hens, Pomegranate, Antioxidant, Interferon, Plasma biochemistry, Egg quality

## Abstract

Pomegranate peel extract is rich in total phenolics with high antioxidant activity. Furthermore, prolonging the laying period of hens is a growing trend that offers both financial and environmental benefits. Therefore, the current study aimed to investigate the potential effects of Pomegranate peel powder extract (PPPE) on aged laying chickens. One hundred twelve ISA White chickens, 80 weeks old, were randomly distributed into 4 groups, with seven replicates of 4 birds each. The first group received a basal diet (control group), which contains 170 mg of crude protein/kg diet with 2800 Kcal ME/kg of diet, while the second, third, and fourth groups received a basal diet supplemented with PPPE at levels of 100, 200, and 300 ppm, respectively. For daily egg mass (EM) per hen, egg production (EP%), feed conversion ratio (FCR), eggshell strength (kg/cm^2^), Haugh unit, total protein (TP), and globulins (G) were significantly (*p* < 0.001) improved by adding 100 ppm of PPPE in laying hens’ diets compared to other treatments. Furthermore, all hens receiving the experimental treatments had the best yolk color, yolk weight, yolk/albumin ratio, shell weight of egg, plasma total cholesterol (Cholest), low-density lipoprotein, T3, and T3/T4 ratio. The shell thickness was recorded as the highest value in birds receiving 200 ppm. Furthermore, laying hens fed a diet supplemented with PPPE100 recorded the highest total antioxidant capacity (TAOC) and phosphorus levels of plasma and the lowest interferon γ (INF-γ) and plasma calcium levels. In conclusion, supplementing PPPE at 100 ppm to the basal diet improved aged laying hens’ performance, quality and strength of eggs, TAOC, (INF-γ), Cholest, LDL, and liver function. In addition, more studies are required to investigate PPPE in laying chickens, especially in the last production phase.

## Introduction

Universal food production faces a worthy challenge, requiring a 60% growth to face the raising demand due to world population growing (FAOSTAT 2013). Eggs considered one of the most inexpensive animal protein sources, so the numbers of layer flock are rapidly raising, aiming to face their needs particularly in developing countries (Dunn 2013). For meeting this increasing request sustainably, latest researches have concentrated on boost lay prolong in layer chicken, to prolong their productive life span up to 100 weeks in the same period without moulting (Bain et al. 2016; Alfonso-Carrillo et al. 2021). Also, Zita et al. (2022) mention that the first period of laying can be stopped or prolong by the moulting, accompanied by other production period, aiming to face depopulation. The prolonging of laying-hens is a tendency and has both financial and environmental benefits through decreasing costs (pullet purchasing) and ameliorating benefits (depopulation) of the breeding (Bain et al. 2016; Molnár et al. 2016). In this regard, Bain et al. (2016) reported that the flock hen numbers (laying and breeding) could be annually reduced 2.5 million chickens, through prolonging the laying period to produce even twenty-five more eggs per hen. At last phase of laying, immune response and production traits in laying hens decline pointedly due to the aging, which have negative effects on the animal hormonal status and metabolism (Attia et al. 2020). Hence, reproductive aging causes huge performance and health reeducation waves; this demands flock disposal, but current global satiation imposes saving as many resources as possible. Therefore, researchers are working towards natural dietary antioxidants to delay aging by amplifying the body’s antioxidant enzymes and reducing free radicals (antioxidant system balance), thereby building up DNA repair and preserving genomic integrity for a longer time and then reducing production costs.

Pomegranate (*Punica granatum*) is considered as edible fruit for human, is extensively cultivated in different countries, including Egypt (El-Hadary and Taha 2020). In recent years, Global production and consumption of pomegranate have augmented greatly due to its various component effects on health of human (Avazeh et al. 2021). Pomegranate peel represents the main part up to 60% then its seed approximately 30% related to fruit weight (Ismail et al. 2012). The powder of pomegranate peel (PPP) is a rich source of phenolic compounds with antioxidant properties, including flavonoids (gallotannins, anthocyanins, gallagyl esters, ellagitannins, hydroxycinnamic acids, hydroxybenzoic acids, and dihydroflavonol), hydrolyzable tannins (gallic acid, ellagic acid, and punicalagin) (Ismail et al. 2012, Ibrahim et al. 2017)). Also, PPP has activity of antibacterial and antifungal (Abdollahzadeh et al. 2011), immunomodulatory (Ross et al. 2001), antioxidant (Al-Zoreky 2009) and various pharmacological functions (Li et al. 2006). Furthermore, pomegranate peel powder extract (PPPE) has hypolipidemic and hypocholesterolemic effects (Belkacem et al. 2010; Ibrahium 2010; Fayed et al. 2012) and acts as an immuno-stimulant (Harikrishnan et al. 2012). The utilization of natural feed additives specially plant origin became more interested for researchers, due to their antimicrobial, antioxidants, antifungal, antibacterial activities (Hassan et al. 2020). More recently, Kamel et al. (2021) showed that the quail diets contain up to 9% of PPP, egg production and the mRNA expression of FSHR and LH-β genes were improved. Adding pomegranate peel powder (PPP) decreased the adverse effects of dexamethasone-induced oxidative stress on egg production. Compared to the positive control, it improved the impact on plasma cholesterol and triglycerides, antioxidative enzymes (SOD, CAT, and GPx), and total antioxidant blood capacity (Eid et al. 2021). Additionally, the 20 g PPP/kg diet supplement boosted the immune response and beneficial bacteria’s proliferation in broiler chickens’ ileal and cecal digesta, while reducing the abundance of pathogenic ones (Rezvani et al. 2016). Thus, the research presumes that dietary supplementation with pomegranate peel powder extract, a natural antioxidant, can support laying hens in their late phase, preserving their production and health state. Therefore, the current study aimed to investigate the effect of pomegranate peel powder extract (ethanolic) on performance, egg quality, thyroid hormones, antioxidant parameters, Interferon γ, lipid and protein profiles of aged laying hens.

## Material and method

### Ethics approval

The current experiment was and approved by Fayoum University Institutional Animal Care and Use Committee (FU-IACUC), Egypt, Approval number: AEC 2326.

### Pomegranate peel extract preparation

Pomegranates (Manfaluti variety) were purchased from the local market, cleaned with distilled water, and peeled. Peels were dried at 40 °C for 48 hours, ground into fine powder, and stored until use. Each 100 g of peels were extracted with 1000 ml of ethanol (80%) in a shaking incubator for 24 hours, filtered with filter paper, and then stored till utilized. According to AOAC (2019), the proximate analysis of pomegranate peel powder extract (PPPE) was assayed. The total phenol, flavonoid, and tannin content of PPPE was determined following the method of Lu et al. (2020), Papaioannou et al. (2020), and Lima et al. (2012), respectively. Anthocyanin was assayed by using a colorimetric technique (Abou-Arab et al. 2011). The 2,2-diphenyl-1-picrylhydrazyl (DPPH) radical-scavenging activity of pomegranate peel extracts was measured according to Yao et al. (2019).

### Birds, design and management

The current trial was performed at the Poultry Research Center, Faculty of Agriculture, Fayoum University, Egypt. One hundred and twelve 80-week-old ISA white hens were randomly distributed into four groups with seven replicates of 4 birds each, each with an initial body weight of 1589.75 ± 9.90 g and a 51.29% egg production rate, for 8 weeks. The first group received a basal diet (control group), which contains 17% crude protein with 2800 Kcal ME/kg of diet, while the second, third, and fourth groups will receive a basal diet plus ethanolic extract of dried pomegranate peel powder at levels of 100, 200, and 300 ppm, respectively. All birds were housed in a laying chicken cage (50 × 50 cm, 625 cm^2^/hen) and exposed to 16 h of light and eight h of dark daily. In addition, water was provided *ad libitum*, and feed was restricted (about 110 g/bird/day).

## Dietary treatments

Feed ingredients and proximate analysis (calculated and analyzed) of the diets are displayed in Table 1. During the trial, the birds were fed a mash diet which was formulated to meet the recommendations of the NRC (1994), considering the age of the hens.

**Table 1:** Feed composition and analysis (calculated and analyzed) of the control diet

Ingredients	%	Calculated Analysis**	
Corn	60.93	CP %	17.00
Gluten 60%	5.00	ME kcl/kg	2,800
Wheat, bran	4.58	Ca %	3.71
Soybean meal 46%	16.88	P non- phytate %	0.35
Soya Oil	1.28	Lysine %	0.88
L-Lysine	0.18	Methionine %	0.45
DL Methionine	0.14	Analyzed ***	
Salt, NaCl	0.35	Crude Protein %	16.71
Calcium carbonate	9.09	Calcium %	3.65
Calcium phosphate, di	1.27	P non- phytate %	0.34
Premix*	0.30	EE %	3.92
Total	100.00	CF %	3.85

* Vitamin and mineral mix contain/3 kg Vit A 10 MIU, vit D_3_ 2.5 MIU, vit E 50 000 mg, Vit K_3_ 3000 mg, vit B_1_ 2000 mg, Vit B_2_ 6 000 mg, Vit B_6_ 5000 mg, Vit B_12_ 20 mg, pantothenic acid 12 000 mg, Niacin 40 000 mg, Biotin 100 mg, Folic acid 1500 mg, Choline chloride 400,000 gm, Selenium 300 mg, Copper 10,000 mg, Iron 30 000 mg, Manganese 80 000 mg, Zinc 80 000 mg, Iodine 1000 mg and CaCO_3_ to 3000 g. **According to NRC (1994). ***According to AOAC 2019)

### Performance traits

Egg number (EN) and Egg mass (EM, g) were daily documented for 8 weeks, then egg production (EP %), average of egg weight (EW, g), feed conversion ratio (FCR) were computed. In addition to, the residual feed (per replicate) was weekly recorded to calculate the feed consumption (FC).

$${\rm{Average\;of\;EW}}\left( {\rm{g}} \right) = {{{\rm{EM}}\left( {{\rm{during\;the\;exprement\;period\;per\;unit}}} \right)} \over {{\rm{EN}}\left( {{\rm{during\;the\;exprement\;period\;per\;unit}}} \right)}}$$

$${\rm{FCR}} = {{{\rm{feed\;cons}}\left( {{\rm{during\;the\;exprement\;period\;per\;unit}}} \right)} \over {{\rm{EM }}\left( {{\rm{during\;the\;exprement\;period\;per\;unit}}} \right)}}$$

$${\rm{EP}}\left( {\rm{\% }} \right) = {{{\rm{EN}}\left( {{\rm{during\;the\;exprement\;period\;per\;unit}}} \right)} \over {{\rm{EM}}\left( {{\rm{during\;the\;exprement\;period\;per\;unit}}} \right)}}$$

### Egg quality

At the end of the trail, 12 eggs per treatment were selected and taken to measure tested characteristics of egg quality.Albumen and Yolk height (mm) were measured. Yolk diameter (cm) was determined by Vernier caliper. Yolk and shell weights (g) were measured by an electronic balance. The albumen weight (g) was calculated by egg weight minus the weight of the yolk and shell. By using the Roche Improved Yolk Color Fan (RYCF), the yolk color was determined. The scale used ranged from 1 (pointing to a light pale color) to 15 (referring to a dark orange color). The thickness of the eggshell (mm) was determined by measuring thickness at the blunt end, sharp end, and middle section of the eggshell, then taking the average. According to Fathi and El-Sahar (1996), the breaking strength of eggshell (kg/cm^2^) was assayed by using the quasi-static compression method. It was carried out at the Poultry Production Department, Faculty of Agriculture, Ain Shams University. The Haugh unit (HU) was calculated using the following equation:

$${\rm{HU}}\,{\rm{ = }}\,{\rm{100}}\,{\rm{*}}\,{\rm{log}}\,{\rm{(H}}\,{\rm{ + }}\,{\rm{7}}{\rm{.57}}\, - \,{\rm{1}}{\rm{.7 W}}{{\rm{ }}^ \wedge }{\rm{0}}{\rm{.37),}}$$

H = albumen height (mm), and W = the egg weight (g).

### Blood sample collection

After 8 weeks of treatment, 20 birds were randomly chosen (5 birds per treatment), from wing vein by using the sterile syringe (Hebrin), the blood samples were collected then transferred to individual test tubes and centrifuged (3500 rpm for 15 minutes). Plasma was isolated and stored at − 20 °C in deep freezer till using.

### Plasma biochemical analyses

All tests were carefully carried out per the manufacturer’s protocols and instructions. Triglycerides (TG), Total cholesterol (Cholest), cholesterol low-density lipoprotein (LDL), and cholesterol high-density Lipoprotein (HDL) were measured according to James (2001). Conforming to Paglia and Valentine (1967), total antioxidant capacity (TAOC) and glutathione peroxidase (GSH-PR) were assayed. Alanine aminotransferase (ALT), alkaline phosphatase (ALP), aspartate aminotransferase (AST), Uric acid, and creatinine (Creat) were estimated by Friedman and Young (2005). According to a commercial kit protocol, total proteins and albumin were assayed, and the difference between TP and Alb was calculated as globulin. Using ELISA kits, we assayed triiodothyronine (T3) and thyroxine (T4). Calcium, phosphorus, and glucose levels were analyzed using a commercial kit from Reactivos GPL in Barcelona, Spain. Interferon γ was also assayed using a product from Invitrogen, Thermo Fisher Scientific Inc.

### Statistical analysis

All data obtained was statistically analyzed by using Infostat program (by the statistics tools, analysis of variance), and the model as follows:

$${{\rm{Y}}_{{\rm{ij}}}}{\rm{ = \mu \;+ \;}}{{\rm{T}}_{\rm{i}}}{\rm{ \;+\; }}{{\rm{e}}_{{\rm{ij}}}}$$

Where: Y_ij_: observation of traits, µ: overall mean, T_i_: treatment effect, e_ij_: random error. To compare among means, the multiple range Duncan test at level of 0.05 was used (Duncan 1955).

## Result and discussion

### Performance traits

Table 2 displayed the effect of PPPE on the performance of aged laying hens. EM, EN%, and FCR significantly (*p* < 0.001) improved by adding 100 ppm of PPPE to laying hens’ diets, while EW and FC were unaffected by treatment. The hens that received diets supplemented with 100 ppm of PPPE recorded the highest egg production percentage (65.4%), followed by 200 and 300 ppm, then a control group. The best values of EM and FCR were recorded when chickens were fed a diet supplemented with 100 ppm of PPPE, followed by PPPE200 and PPPE300, then the control group. This indicates that the values of EM and FCR improved as the levels of PPPE in the supplemented diets decreased.

**Table 2:** Effect of pomegranate peel powder extract on performance of aged laying hens

Treatments Item	Control	PPPE_100_	PPPE_200_	PPPE_300_	Pooled SEM	P value
EP%	49.4^c^	65.4^a^	59.1 ^b^	56.1^b^	1.58	< 0.001
EW (g)	63.2	62.2	62.1	62.9	1.19	0.88
EM(g/h/d)	31.2^c^	40.6^a^	36.7^b^	35.2^b^	1.12	< 0.001
F Cons (g)	109	110	109	109	1.5	0.92
FCR	3.53^a^	2.73^c^	2.97^bc^	3.12^b^	0.106	< 0.001

PPPE100, PPPE200 and PPPE300: a basal diet plus ethanolic extract of pomegranate peel powder at levels 100, 200 and 300 ppm respectively. SEM: Stander Error for means. EM(g/h/d): egg mass (gram/ hen/day), EP%: egg production percentage, EW: Average of egg weight, FCons: feed consumption. FCR: feed conversion ratio. a−c Means within the same row with different superscript

### Egg quality

As shown in Table 3, the adding PPPE to diets significantly (*p* < 0.016) affected the characteristics of yolk, albumin, shell, and yolk/albumin ratio in chickens’ egg. Hens receiving PPPE had the highest yolk color, yolk weight, and yolk/albumin ratio. The highest albumin height and haugh unit were recorded in groups receiving PPPE100 and PPPE300 compared to PPPE200 and control. Albumin weight decreased in chickens receiving PPPE100 and PPPE300, also, birds receiving PPPE levels had higher shell weight. The thickness was highest in birds receiving PPPE200, followed by PPPE100 and PPPE300. The shell strength was recorded the best value in group received PPPE100 compared to PPPE300 and PPPE200 then control, being 5.29, 4.51, 4.90 and 3.96, respectively.

**Table 3:** Effect of pomegranate peel powder extract on egg quality of aged laying hens

Treatments Item	Control	PPPE_100_	PPPE_200_	PPPE_300_	Pooled SEM	P value
Yolk traits						
Colour fan score	9.00^b^	10.3^a^	10.7 ^a^	10.8 ^a^	0.323	< 0.001
Weight%	26.2^b^	28.0^a^	25.8^b^	27.2^ab^	0.49	0.017
Yolk/Albumen ratio	40.6^bc^	45.0^a^	40.1^c^	43.5^ab^	1.12	0.009
Albumen traits						
Height (mm)	8.30^b^	9.24^a^	9.44^a^	8.57^b^	0.129	< 0.001
Weight%	64.9^a^	62.3^c^	64.4^ab^	62.7^bc^	0.61	0.008
Haugh unit	90.6^b^	95.2^a^	95.8^a^	91.2^b^	0.75	< 0.001
Shell traits						
Weight%	8.83^b^	9.76^a^	9.75^a^	10.1^a^	0.245	0.005
Thickness (µm)	29.0^c^	32.3^b^	34.2^a^	32.2^b^	0.55	< 0.001
Breaking strength (kg/cm^2^)	3.96^c^	5.29^a^	4.51^bc^	4.90^ab^	0.233	0.004

PPPE100, PPPE200 and PPPE300: a basal diet plus ethanolic extract of pomegranate peel powder at levels 100, 200 and 300 ppm respectively. SEM: Stander error for means. a−c Means within the same row with different superscript

### Plasma lipid, protein profile and glucose values

The results were presented in Table 4 revealed that the adding PPPE significantly (*p* < 0.001) decreased Cholest and LDL in aged layer hens, while TG and HDL were not significant effect. Furthermore, the best effect (decreased values) was observed in the group treated by all PPPE levels for Cholest and LDL. Hens fed a diet with PPPE 100 have the highest TP (7.81) and Glo (5.64) values followed by the other groups. While, basal diets were recorded the lower Alb value (2.72). Chickens fed diets with PPPE levels 300 and 200 ppm showed a significant (*p* < 0.017) decrease in plasma glucose levels compared to other groups (control and PPPE100).

**Table 4:** Effect of pomegranate peel powder extract on the plasma lipid and protein profile of aged laying hens

Treatments Item	Control	PPPE_100_	PPPE_200_	PPPE_300_	Pooled SEM	P value
Plasma lipid profile						
TG (mg/dl)	1286	1634	1458	1511	116	0.24
CHOL (mg/dl)	354^a^	232^b^	192^b^	225^b^	15.4	< 0.001
HDL (mg/dl)	25.3	21.8	21.2	22.8	1.42	0.22
LDL (mg/dl)	222^a^	129^b^	102^b^	108^b^	10.3	< 0.001
Plasma protein metabolism						
Total protein (g/dl)	7.32^b^	7.81^a^	7.15^b^	6.94^b^	0.145	0.005
Albumin(Alb) (g/dl)	2.72^a^	2.16^b^	2.13^b^	2.27^b^	0.063	< 0.001
Globulins (G) (g/dl)	4.60^b^	5.64^a^	5.02^b^	4.68^b^	0.156	< 0.001
Alb/G	0.59^a^	0.38^c^	0.43^bc^	0.49^b^	0.023	< 0.001
Glucose						
Glucose (mg/dl)	262.6^a^	225.0^ab^	210.8^b^	196.0^b^	13.34	0.017

PPPE100, PPPE200 and PPPE300: a basal diet plus ethanolic extract of pomegranate peel powder at levels 100, 200 and 300 ppm respectively. SEM: Stander error for means. CHOL: cholesterol concentration. TG (mg/dl): triglyceride concentration. HDL: high density lipoprotein. LDL: low density lipoprotein. a−c Means within the same row with different superscript

### Liver and kidney function and thyroid hormones

Data in Table 5 showed that ALT, Creat, ALP, T3, T4, and T3/T4 ratio levels were significantly (*p* < 0.001, except T3 *p* < 0.03) influenced by treatments, while AST and uric acid were not affected by the treatments. Where the laying hens fed a diet supplemented with PPPE300 recorded the lowest values of ALT and ALP, and the highest creatinine in plasma. The birds that received PPPE in their diets had the highest concentration of T4 and T3/T4 ratio, while those that received PPPE 100 had the lowest level of T3 compared to other treatments.

**Table 5:** Effect of pomegranate peel powder extract on liver and kidney function and thyroid hormones of aged laying hens

Treatments Item	Control	PPPE_100_	PPPE_200_	PPPE_300_	Pooled SEM	P value
Liver function						
ALT (U/L)	17.8^a^	14.0^b^	12.4^bc^	10.6^c^	0.98	< 0.001
AST (U/L)	234	237	234	227	18.0	0.98
ALP (U/L)	365.6^ab^	336.8^bc^	417.6^a^	269.8^c^	23.80	0.004
Kidney function						
Uric acid (mg/dl)	7.14	8.24	6.94	7.82	0.642	0.47
Creatinine (mg/dl)	0.52^c^	0.72^a^	0.75^a^	0.73^b^	0.029	< 0.001
Thyroid hormones						
T3 (ng/ml)	4.37^b^	5.05^a^	4.56^b^	4.47^b^	0.154	0.031
T4 (ng/ml)	25.0 ^a^	21.7^b^	21.8^b^	21.1^b^	0.49	< 0.001
T3/T4 ratio	0.18^b^	0.23^a^	0.21^a^	0.21^a^	0.007	< 0.001

PPPE100, PPPE200 and PPPE300: a basal diet plus ethanolic extract of pomegranate peel powder at levels 100, 200 and 300 ppm respectively. SEM: Stander error for means. ALT: Alanine aminotransferase. AST: Aspartate aminotransferase. ALP: Alkaline phosphatase. a−c Means within the same row with different superscript

### Antioxidant, anti-inflammatory parameters, and minerals of plasma

As presented in Table 6, TAOC was significantly (*p* < 0.001) promoted when hens were supplemented with PPPE at levels 100 and 300 compared to other groups, while GSH-PR levels were insignificantly affected by all treatments. IFN-γ was significantly (*p* < 0.001) affected by PPPE, where the lowest values were recorded with 100 ppm, followed by 300 and 200, then the control group. Plasma phosphorus levels were significantly (*p* < 0.001) increased by supplementing with PPPE_300_, followed by PPPE_200_ and PPPE_100_, then control, being 6.98, 5.97, 5.91, and 5.43, respectively. Birds received a control diet, and diets containing PPPE _300_ had higher plasma calcium levels compared to other groups.

**Table 6:** Effect of pomegranate peel powder extract on antioxidant, anti-inflammatory, and mineral levels of aged laying hens

Treatments Traits	Control	PPPE_100_	PPPE_200_	PPPE_300_	Pooled SEM	*P value*
Antioxidant enzymes						
TAOC (mmol/L)	0.10^c^	0.13^a^	0.12^b^	0.13^ab^	0.004	< 0.001
GSH-PR (U/L)	274	291	304	289	15.24	0.60
Anti-inflammatory						
Interferon γ (ng/ml)	40.6^a^	30.1^c^	35.7^b^	35.3^b^	0.67	< 0.001
Minerals						
Ca t (mg/dl)	29.8^a^	26.1 ^c^	27.3 ^bc^	28.6 ^ab^	0.48	< 0.001
Phos (mg/dl)	5.43^c^	5.91^b^	5.97^b^	6.98^a^	0.124	< 0.001

PPPE100, PPPE200 and PPPE300: a basal diet plus ethanolic extract of pomegranate peel powder at levels 100, 200 and 300 ppm respectively. SEM: Stander error for means. TAOC: total antioxidant capacity. GSH-PR: Glutathione peroxidase. Ca t: plasma calcium concentration. Phos: plasma Phosphorus concentration. a−c Means within the same row with different superscript

**Table 7:** Proximate composition and bioactive components of pomegranate peel extract used in the experiment

Item	Unit	Value
Chemical composition		
Dry matter	(mg /g)	960.15
Crude protein	(mg /g)	81.21
Crude fiber	(mg /g)	215.13
Ash	(mg /g)	55.31
Ether extract	(mg /g)	28.99
Nitrogen free extract	(mg /g)	99.09
Bioactive components		
Extraction yield	% (w/w)	35.15
Total phenolic	(mg gae/g)	455.63
Total flavonoid	(mg qe/g)	240.31
Total tannins	(mg tannin/g)	349.15
Anthocyanin	(μg C3G/g)	45.76
DPPH	%	89.26

mg: mille gram. g: gram, W: weight, GAE: Gallic acid equivalents, QE: quercetin equivalent, μg: microgram, C3G: Cyanidin-3-o-glucoside, DPPH: 2,2-diphenyl-1-picrylhydrazyl

## Discussion

The available data about aging hens (particularly after 80 weeks of age) is limited, including blood antioxidants, thyroid hormones, sexual hormones, lipid and protein profiles, and liver and kidney function. The utilization of natural feed additives, especially those of plant origin, became more interesting for researchers due to their antimicrobial, antioxidant, antifungal, and antibacterial activities. Our results may be attributed to the pomegranate peel powder extract being characterized by having active ingredients including; total phenolic, total flavonoid, Total tannins and DPPH being 455.63 (mg GAE/g), 240.31 (mg QE/g), 349.15 (mg tannin/g) and 89.26%, respectively, as detected in the PPPE (Table 7). So, the PPPE has high capacity of antioxidant, regarding its activity of scavenging and protective versus peroxyl radicals, hydroxyls, and superoxide anions (Kishawy et al. 2019). Furthermore, the properties of antioxidants can protect the mucosa of the intestine against pathogens and oxidative damage by stimulating the enzymatic activity of the gastrointestinal tract, thus enhancing digestion and metabolism (Abbas et al. 2017; Hassan et al. 2020; Kamel et al. 2021; Hongrui et al. 2025). The production traits improvements may be due to the PPP improving mRNA expression of FSHR and LH-β genes in laying quails (Kamel et al. 2021). The antioxidant polyphenols in Grape seed extract can regulate diverse signaling pathways involved in oxidative stress, inflammation, reproductive hormones, apoptosis, steroid hormone receptors, and cell growth (Kohut et al. 2024). So, the improved performance may be due to the PPE antioxidant effect, which protects the sexual hormone receptors. In other words, our result suggests this improvement is due to improved health status and reduced protein consumed in immunity protein synthesis (immune cost): The inflammatory sequence of pro-inflammatory proteins has decreased (as shown in Table 6), and the A/G ratio has also decreased (as shown in Table 4); consequently, all body proteins are now directed towards egg production (Richards et al. 2005). Our findings agree with Hongrui et al. (2025), who concluded that the EP%, egg weight, and FCR were promoted by the addition of pomegranate peel polyphenols (PPPs) in diets of laying chickens. In this regard, Abbas et al. (2017), and Kamel et al. (2021) found that the egg production traits (EP%, EM, and FCR) were improved by adding PPP in quails’ diets. Furthermore, the results obtained by Eid et al. (2021) showed that a diet supplemented with 4 g of PPP/kg alleviated Dexamethasone negative effect on egg production% of laying chickens. Unfortunately, no previous studies related to the pomegranate peel powder extract of aged laying hens have been conducted.

For egg quality, the current results agree with Hongrui et al. (2025) who showed that the HU, breaking strength, egg thickness and albumen height were (*p* < 0.05) significantly improved when laying hens received PPPs. Eggshell thickness and strength were greatly influenced by calcium, which is the main building block (Vijay et al. 2021). In addition to Hongrui et al. (2025), who concluded that the calcium apparent digestibility and absorption were significantly improved when hens were fed a diet supplemented with PPPs. Therefore, eggshell weight, egg thickness, and breaking strength were enhanced by the addition of PPPE, which improved calcium apparent digestibility. The PPPE improved HU, yolk and albumen weight and yolk/Alb ratio, which may be due to enhance nutrient apparent digestibility.

Regarding lipid profile, our findings indicated that plasma Chol and LDL concentration lowered by adding of PPPE to basal diet compared to untreated group, this change being due to the positive effect of pomegranate on lipid metabolism (Hou et al. 2019). Also, the pomegranate peel has exerted hypolipidemic and hypocholesterolemic impacts and intervened with digestion, absorption, and metabolism of lipids (Lei et al. 2007; Hossin 2009; Fayed et al. 2012; Kishawy et al. 2016). Furthermore, Lv et al. (2016) mention that the polyphenols, ellagic and punicalagin, that found in PPP, improve cholesterol metabolism through enhancing total production of bile acid in the cells of the liver. The current results agree with Yassein et al. (2015) they found that blood cholesterol was decreased by pomegranate by-product to broiler diets compared to un-supplemented treated. Also, Eid et al. (2021) found that plasma cholesterol was significantly decreased and triglyceride was not affected when the laying chickens were fed a diet supplemented with PPP. Similar results obtained by Soliman and Kamel (2020) who reported that inclusion of 10gPPP/diet lead to decrease of cholesterol and LDL concentration without effect on TG and HDL concentration in plasma. Furthermore, Kishawy et al. (2019) showed that the broiler diet supplemented with PPE, either with linseed oil or with soybean oil, at 0.05% or 0.1%, led to a decrease in TG, Chol, and LDL concentration and an increase in HDL concentration in broiler serum. With laying quails Yassein et al. (2015) found that a diet containing PPP at levels 1 or 1.5%, and the plasma cholesterol and LDL concentration were decreased. Regarding protein profile, the current results agree with El-Rayes et al. (2023) who mention that the quails received PPP (300, 600 and 900 g/ton) in diets, the TP and globulins were significantly increased, suggesting that serum TP and Alb enhancement had been associated to promoted protein digestibility (Oliveira et al. 2010). Also, this result agree with Yassein et al. (2015) who found that the plasma glucose level was decreased when laying quails fed on diets containing pomegranate peel powder at 10 or 15 PPP/kg diet.

The findings of the present study agree with El-Rayes et al. (2023). They suggested that diets containing PPP have a potent hepatoprotective impact due to their content of phenolic and antioxidant components as anthocyanidins, punicalagin, flavanol, gallic acid, fatty acids, flavanones, catechin, quercetin, rutin, flavonols, flavones. The current results agree with Hongrui et al. (2025), who found that both of ALT and ALP were improved when laying hens received PPPs. On the same line, Abbas et al. (2017) found that inclusion of PPP into laying quail diets led to decrease of ALT concentration without effect on AST. They suggest that PPPs have the capability to preserve the liver. Our results disagree with Yassein et al. (2015) who mention that laying quails fed a diet contain a PPP, the AST, uric acid and creatinine were significantly decreased. For uric acid, the present findings agree with Kamel et al. (2021) who reported that the uric acid was not affected by adding of PPP into diets of laying quails. As reported in Table 7, the PPPE contain a pigment, therefore the plasma creatinine concentration was increased with PPPE administration to hen diets. This result agrees with Weber et al. (2013) who reported that plasma creatinine concentration was augmented with Canthaxanthin (as a pigment) adding in diets of chickens. This increasing of creatinine may be regarded to pigmented plasma interferences with photometric determination, since the creatinine analytical method is based on the reaction of Jaffe where creatinine reacting with picrate in an alkaline medium yields an orange-stained product (Weber et al. 2013).

Thyroid hormones (T3 and T4) are considered an important factor for avian reproductive processes, the seasonality initiation of the reproductive cycle, and the energy-requiring processes, without competing with reproduction in energy-demanding (McNabb and Darras 2015). In addition, Wentworth and Ringer (1986) mention that the aging tends to decline the thyroid hormone secretion rate. In context, Badr El Dine et al. (2017) showed that the thyroid hormone impairment may be attributed to the thyroid follicular cells’ decline, which is regarded to oxidative stress. Hayouni et al. (2011); Dos Santos et al. (2016) found that pomegranate peel extracts have a crucial function in anti-aging. The effects of antioxidants in the pomegranate peel were detected, which relate to its high polyphenol content. It is rich in ellagitannins (punicalagin and its derivatives), which may contribute to its strong antioxidant potential (Mohamed and Mabrok 2021; Sayed et al. 2022). Therefore, it has a protective effect, which leads to thyroid function improvement and oxidative stress reduction, and apoptosis (El-Din et al. 2023). Pomegranate extracts can remove anions of free superoxide and hydroxyl radicals and prevent the peroxidation of lipids and oxidative stress (Rosenblat et al. 2006; Kanatt et al. 2010). For T3, our findings agree with Hongrui et al. (2025), who found that serum T3 concentration was significantly increased when hens were fed a diet with pomegranate peel polyphenols. Inflammatory response is considered to be directly caused by oxidative stress (Guzik and Touyz 2017; Wang et al. 2017).

Regarding antioxidant parameters, our findings agree with (Abdel-Wahab and Mosad 2018), who found that GSH-PR had no significant effect by adding of PPP (0.5, 1.0, and 1.5%) to quails’ diet. The results obtained by Sari et al. (2012) indicated that the higher values of calcium digestibility led to decreasing of plasma calcium levels and (Hongrui et al. 2025) mention that the digestibility of calcium was increased when hens received PPPs in their diets. So, the plasma calcium concentration in the current study was decreased when laying hens fed on a diet supplemented with PPPE. The present result agree with Liu et al. (2022) who concluded that IFN-γ was significantly declined when aged laying hens received a diet supplemented with antioxidant (lipoamide). Antioxidants (selenium and various phytogenic compounds) significantly decrease oxidative stress in laying hens. The cellular damage reduction means that the immune system does not need to mount as strong an inflammatory response, naturally leading to decreased production of pro-inflammatory cytokines, including interferons (Schmucker et al. 2021; Li et al. 2022; Wang et al. 2024). Moreover, antioxidants stimulate the Nrf2 signaling pathway, which upregulates protective enzymes and decreases gene expression of inflammatory molecules. This pathway activation helps support cellular homeostasis without requiring high levels of inflammatory mediators (Griela et al. 2021; Surai et al. 2019). The interferon levels decrease during high egg production periods, when laying hens receive antioxidant supplementation in their diets. This decrease is due to fundamental physiological trade-offs between immune function and reproductive performance. Egg production and immune responses are energetically expensive processes that compete for limited resources within the bird’s metabolic system (French et al. 2007).

## Conclusion

The addition of 100 ppm of PPPE to the diets of aged laying hens improved EM, EP%, FCR, eggshell strength (kg/cm2), Haugh units, TP, and G. All hens receiving PPPE at concentrations of 100, 200, and 300 ppm exhibited the best yolk color, yolk weight, yolk/albumin ratio, shell weight, plasma cholesterol, LDL, T3, and T3/T4 ratio. The highest shell thickness was recorded in birds receiving PPPE at 200 ppm. Laying hens fed a diet supplemented with 100 ppm of PPPE showed the highest total antioxidant capacity (TAOC) and phosphorus levels in plasma and the lowest levels of interferon γ (INF-γ) and calcium in plasma. Furthermore, additional studies are needed to investigate the effects of PPPE in laying chickens, particularly during the last production phase.

## Data Availability

The data that support the finding of this study are available from the corresponding author upon reasonable request.
